# Persistence in Problematic Internet Use—A Systematic Review and Meta-Analysis

**DOI:** 10.3389/fsoc.2020.00030

**Published:** 2020-05-15

**Authors:** Daniel Dahl, Karin Helmersson Bergmark

**Affiliations:** Department of Sociology, Faculty of Social Sciences, Stockholm University, Stockholm, Sweden

**Keywords:** gaming disorder, internet addiction, problematic internet use, persistence, longitudinal, panel, systematic review, meta-analysis

## Abstract

**Background and Aims:** Problematic internet use, internet addiction, and internet gaming disorder all describe a global phenomenon where individuals have trouble limiting their use of internet to such an extent that their use has negative consequences. Past systematic reviews and meta-analyses have focused on estimating prevalence, but there has been no comprehensive research synthesis of the trajectory of the problem. The research objective was to create a pooled estimate of the persistence of problematic internet use. This review included studies using a longitudinal panel data design with a follow-up of at least a year. Studies had to be published before the end of the year 2017, in peer-reviewed academic journals, using English language. Samples from populations in any country were accepted, given they were of acceptable quality.

**Methods:** A literature search was conducted in Web of Science, Pro Quest, and Scopus. Several definitions of problematic internet use were included. Inverse-variance, random-effect meta-analysis was used to estimate weighted summary means of persistence. Attrition and selection bias was investigated using pre-specified tools, and heterogeneity was assessed in subgroup analysis.

**Results:** Nine studies fit the criteria, all using samples from Asian or Western countries. The aggregate estimate for 1-year persistence it was 50% (CI: 40–61%), but results were heterogeneous. Prevalence and persistence estimates were correlated and generally higher in Asian countries. Methodological differences only explain part of the heterogeneity.

**Conclusion:** All included studies found individuals with persistent problems, but the between-studies variation is substantial.

## Introduction

Ever since the 1990s, worries have been raised about the consequences of spending too much time on internet-enabled devices. Due to its similarities with compulsive disorders or addiction, the American Psychiatric Association, APA, has proposed Internet Gaming Disorder (IGD) as a future introduction in the DSM (American Psychiatric Association, 2013), and the WHO now includes gaming disorder as a non-substance addictive disorder in the latest edition of ICD-11 (World Health Organization, 2018).

Heavy internet use is part of a cultural shift in society, where our lives become more dominated by internet. Gaming, chat rooms, and other online venues can act as alternative social arenas—and not only for those who are less likely to interact in traditional settings such as bars, clubs, or parties. Findings even indicate that some heavy use of internet could benefit individuals (Chou et al., 2005). Bergmark et al. (2011) pose that, for some internet activities there is no clear-cut line between lifestyle and addiction, seconded by Ng and Wiemer-Hastings' (2005) proposal that many game players' perspective on social life and lifestyles can differ from those who do not play. In a chapter based on case studies from the early 2000s, Griffiths (2008) emphasizes that many spend excessive time playing games on internet-enabled devices without suffering negative consequences, however, acknowledging that video game playing is sometimes used to counteract offline life deficiencies, problems, or dissatisfaction.

The past decades have seen a surge in literature on the subject, and while the existence of a problem is well-documented, some aspects are yet to be affirmed: several attempts at establishing an expected prevalence level of problematic internet use have delivered varying results, ranging between 0.7 and 27.5% (Mihara and Higuchi, 2017). A cross-country European study found prevalence rates spanning between 0.6 and 2.6% (Müller et al., 2015). Cheng and Li (2014) found prevalence levels in Asian countries around 7.1%, which was similar to North America (8%) and South-East Europe (6.1%). These differences, however, were not supported by Mihara and Higuchi (2017), nor Feng et al. (2017) who found no systematic differences in prevalence according to region.

Although prevalence is well-covered in primary research as well as reviews, a related aspect lacks a proper capitulation: does problematic internet use tend to be persistent or transitory (Hellman et al., 2013)? General definitions of addiction require that it causes serious problems, failure(s) to stop it, and persistence over time, often at least a year (World Health Organization, 2018). This holds true in the ICD-11 (World Health Organization, 2018), as well as the proposed DSM-5 criteria (American Psychiatric Association, 2013).

Mihara and Higuchi's (2017) systematic review of longitudinal studies found reports that gaming disorder and similar conditions were persistent, as well as the opposite. They suffice with a narrative review, without a meta-analysis, and do not investigate into factors that can provide a better understanding between the differences in results between the studies. Thus, the research objective is to examine if there is evidence to support that problematic internet use is best viewed as a persistent^1^ condition. This will be performed through systematic review and meta-analysis of all international available literature on the subject, complemented with subgroup analysis of some factors which may help explain divergent results.

Until the ICD-11 introduction, there was no gold standard for measuring problematic internet use. Efforts to identify all existing measures have found a plethora of diagnostic scales (Laconi et al., 2014), based on criteria for pathological gambling, substance dependence, or a combination of the both (Kuss and Griffiths, 2012; Kuss et al., 2014).

Although the DSM-5 and ICD-11 single out gaming as their main focus, it is predated by umbrella terms, such as internet addiction or problematic internet use, covering several types of excessive behaviors which are mediated by the internet-enabled devices, including, but not limited to gaming, and excluding already established disorders or problems, like gambling and sexual compulsions, since they are too dissimilar (Pawlikowski et al., 2014). The terms internet addiction and problematic internet use are blunt, and convey a lack of understanding of how to best perceive life online, and it is necessary that the analysis of problems related to life online be divided into meaningful empirical categories. This is also supported by a growing research consensus (Starcevic and Billieux, 2017; Van Rooij et al., 2017). This study uses problematic internet use (Spada, 2014) as the umbrella term for internet addiction, gaming disorder, pathological gaming, and other concepts that have been used by researchers to describe the same problem: individuals having trouble limiting their use of internet to such an extent that their use has negative consequences. If other terms are employed, it is to correctly mirror the terminology used in referenced studies. By using this inexact construct, we aim to include studies using both specific and more general definitions. Since the research field is still young and several definitions have been used by scholars, a single specific definition would lead to a very sparse review, missing studies that might be useful.

The initiation of overuse of internet and gaming can serve as an escape from other problems or failures in life (Kardefelt-Winther, 2014; Ballabio et al., 2017). Although some may use internet activities of various sorts to successfully alleviate stress or anxiety, and find community among other gamers or online contacts, it can also exacerbate existing problems. This has been described by several scholars (King and Delfabbro, 2018) as a *vicious circle*, where individuals continue to withdraw from outside life, further increasing their ill-being and moving further away from solving underlying problems or conditions.

Several studies have found co-occurence between problematic internet use and other indicators of suffering, such as anxiety and depression, but also fewer social activities and lower school grades (González-Bueso et al., 2018). De Leo and Wulfert (2013) suggest that problematic internet use is more associated with depression and being socially anxious than is substance abuse, Kuss and Griffiths (2012) found dysfunctional socialization and personal dissatisfaction to be risk factors for problematic internet use, and among high-frequency online gamers, conscientiousness, and extraversion appeared as protective factors against developing problematic behavior. Kuss et al. (2014) review highlighted various psychosocial problems that could be indicative of excessive internet use as dysfunctional coping for other stressors, such as bad school performance or family problems. Stepanikova et al. (2010) found time spent browsing online to be positively correlated to loneliness and correlate negatively with life satisfaction, indicating that it might insufficiently compensate for offline social interaction.

Mihara and Higuchi's (2017) review identified gender differences in almost all cross-sectional studies: internet gaming disorder is generally more common among males than females. Gender differences may, however, be accredited to systematic differences in internet use habits, which have been documented in both the United Kingdom and China (Li and Kirkup, 2007).

In a review article, Saunders et al. (2017) stress that studies using samples from Asian populations tend to give higher prevalence than in samples from Western countries. Block (2008) argues that there are cultural differences in how and where internet-enabled devices are used in the United States and Asia, where in the former case the use often takes place at one's home, while in the latter, most activity takes place in internet cafés.

Family background is relevant when studying adolescent trajectories, health and well-being (Hjern, 2006; Vinnerljung et al., 2007), although research on problematic internet use and family background is still underdeveloped. One finding by Lai and Kwan (2017) using a Hong Kong sample, was that having a father with higher education, and a family with a high income was associated with problematic internet use, while a higher educated mother seemed protective. Similar results were presented in a Turkish study (Ak et al., 2013).

Van Rooij and Kardefelt-Winther's (2017) criticize that general samples in functional populations risk missing those who have real functional impairment due to their problems, since they tend to not be reached or engage in filling out survey questions. Thus, there is a risk of attrition, which may largely be made up of the studies' real target group.

## Data and Methods

A systematic review synthesizes evidence of a field. It is useful when a research question may be answered by already published studies. A systematic review contrasts traditional reviews, where qualitative analysis, cherry-picking, and/or vote-counting of quantitative studies can bias results and conclusions. Instead, the core of systematic reviews is a strive for reproducibility, efforts to find all available research, and problematizing quality of findings (Petticrew and Roberts, 2006, p. 2). This review is based on guidelines proposed by the Joanna Briggs Institute's Reviewers Manual for Systematic Reviews of Prevalence and Incidence (Short: JBI manual; Munn et al., 2017). It is particularly tailored toward the systematic review and meta-analysis of prevalence studies, by an institute specializing in the broadening of research synthesis methods. The JBI manual builds on the established systematic review-principles of transparency, rigor, and study quality appreciation proposed in the PRISMA (Moher et al., 2009) and STROBE (Von Elm et al., 2007) statements, where PRISMA dictates the gold standard for evaluations of randomized trials and STROBE is tailored toward reporting on epidemiological studies.

Three academic databases were used to find studies: Scopus, Web of Science, and ProQuest. The search string contained several phrases for problematic internet use, such as internet addiction and gaming disorder, as well as various synonyms indicating a longitudinal study design. The contents of the search strings were the same^2^ for all three databases, however, the syntax varied slightly due to the databases different standards.

### Inclusion Criteria

Below follows the criteria for studies to be considered for inclusion in the review. Studies had to utilize a survey design estimate prevalence of problematic internet use (or another equivalent label) at two time points, employ a panel design that allows the same individuals to be followed, be published in a peer-reviewed journal before the end of 2017 (no lower time limit is set). The published studies had to be accessible in full-text in English. No other requirements were set at this stage.

This review includes research on problematic internet use, internet addiction, gaming disorder, pathological gaming, and any similar definition. A sole focus on research using the term *gaming disorder* would make the scope too narrow, since the research field is still young and gaming problems have also been defined as problematic internet use or internet addiction.

Treatment studies are excluded, as well as studies focusing on brain monitoring and neural responses, and studies using qualitative methods. Although covering the same topic, they represent areas in the research field that would warrant their own reviews with different methodological assessments. Gray literature is not included: This review does not seek for an effect or a correlation, which is where worries about publication bias are the largest. Furthermore, prevalence and persistence are often presented as descriptive statistics. Thus, it should not be a defining factor in the decision of publishing a study.

### Study Selection

**Figure 1:**
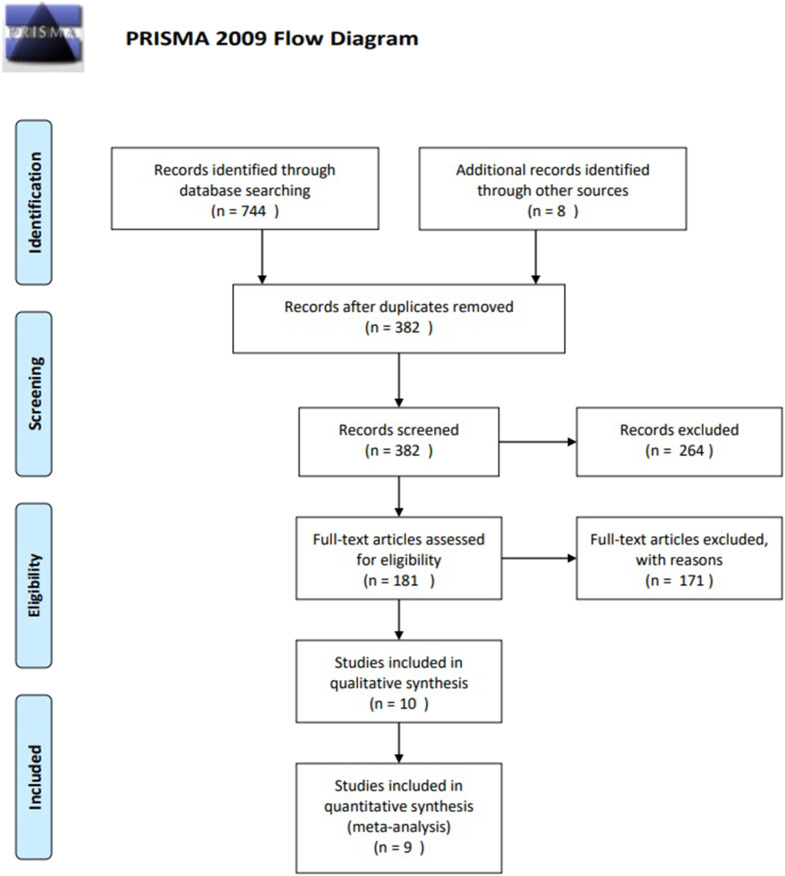
PRISMA flow chart of the study selection process.

See Figure 1 to follow the study selection process (adapted from Moher et al., 2009). Search results were downloaded from the database searches and organized using a reference manager, Endnote X7 (*n* = 744). Double publications were removed, of which the studies with richest data were kept. Then, titles and abstracts of studies were read.

The search was complemented by a process of hand searching in three steps. First, three important journals, using their websites' search functions: *Addiction, Computers in Human Behavior* and *Cyberpsychology & Behavior*. The journals have slightly different foci which all intersect the subject of this paper. Then followed hand searching of the reviews most closely related to this review (Kuss et al., 2014; Anderson et al., 2017; Feng et al., 2017; Mihara and Higuchi, 2017), and third, hand searching of the references in a random sample of ten of the database generated results. In total searching generated eight studies with titles or abstract containing information that indicated they should be considered for inclusion in the review.

After hand searching and duplicates were removed, titles and abstracts of *n* = 382 studies were screened, *n* = 181 studies were assessed in full-text for eligibility, of which *n* = 32 studies measured prevalence at least twice. Eleven studies provided enough information to make it to quality assessment. Finally, *n* = 9 (Ko et al., 2007, 2014; Gentile et al., 2011; Van Rooij et al., 2011; Chang et al., 2014; Scharkow et al., 2014; Thege et al., 2015; Strittmatter et al., 2016; Lau et al., 2017) both contained sufficient data and were considered as being of high enough standard to be included in the review and meta-analysis.

There is no firm rule to decide for how many studies are appropriate in a meta-analysis, and there is generally a lack of directions for conducting meta-analyses on prevalence studies. It is certain that the lower number of studies included in the meta-analysis, the lower will the statistical power be. However, as argued by Borenstein et al. (2009 p. 357), intuitive summaries such as vote counting may be even worse. A way forward is to perform a meta-analysis with an awareness of the limitations, to avoid false certainty in the results. Therefore, this meta-analysis is complemented by sub-group analysis as well as discussions on limitations of the results. Furthermore, it is important to acknowledge that the contribution of a systematic review lies not only in the summary estimate, but also in the number of studies identified and the collection of their different traits.

### Quality Assessment

This systematic review and meta-analysis used the Joanna Briggs Institute's Critical Appraisal Checklist (Munn et al., 2014, 2017) as guidance, along with Hoy et al.'s risk of bias tool 2012. None of the checklists employ a point scale with a specific cut-off for inclusion or exclusion, but help researchers discover strengths and weaknesses in studies, to make an informed decision on which studies are good enough to be included in the systematic review.

### Analytic Strategy

The meta-analysis used Stata 15.1, and the *metaprop* program, which is especially tailored for meta-analysis of binomial data (Nyaga et al., 2014). The objective was to produce weighted means of persistence and compare them in a forest plot.

Prevalence is defined as the proportion of individuals in a sample that has been diagnosed with problematic internet use and is expressed as a percentage of the respondents which could be matched after 1 year. Categorical persistence, being the outcome in the meta-analysis, is measured as those who fulfill criteria for problematic internet use at T1 and T1 + 1 year. It is the percentage of the subgroup of those who fulfilled the criteria (prevalence) at T1. Persistence after baseline +1 year was used in the meta-analysis. One study (Gentile et al., 2011) was 2 years long and used a method where all data collections were used to generate latent classes. Thus, persistence for the 1-year follow-up is not available, and data from the 2-year follow-up is used in the meta-analysis.

A random-effects, inverse-variance model was used since both statistical and clinical heterogeneity was expected, due to results of previous reviews. A score confidence interval is used so even small sample sizes can be accounted for, as well as a Freeman-Tukey double arcsine transformation of proportions to make normal approximation significance tests more applicable (Nyaga et al., 2014). The I^2^ statistic was used to describe how much of the between-studies variation that is due to heterogeneity and not chance (Higgins et al., 2003). The true variation is, of course, unknown.

In a subgroup analysis, means were also computed for a priori defined subgroups which are presented together to provide a visual account of the dispersion of heterogeneity. The following subgroups were generated through findings in previous research and methodological assumptions: (i) Geographic location (ii) Scale used (iii) Focus: general internet use or gaming. Furthermore, it was investigated if there is a relationship between persistence and prevalence. Since persistence is depends on the size ofn prevalence, does this mean that a high prevalence measure tends to give a high persistence measure?

Finally, a narrative review collected (i) information on the scales used and if there seems to be a risk that differences in cut-offs may affect the estimates (ii) if studies focusing on adolescents report different persistence estimates than studies focusing on adults or children (iii) if the studies have a gender balance in their samples, and (iv) if the studies report any systematic variation in persistence when including psychosocial or socio-economic measures.

## Results

### Study Characteristics

Characteristics and basic information about the nine studies included in the review can be found in Table 1. All studies were published in 2007 or later, using data collected between 2003 and 2014. Four studies were longer than 1 year. T1 sample sizes varied between 517 and 9,666. Follow up-rates (the proportion of matched individuals between waves) were high overall, over 65%, but two studies were remarkably low, with follow-up rates at 20% (Scharkow et al., 2014), and 35% (Strittmatter et al., 2016).

**Table 1:** Data collection.

**First author**	**Chang et al. (2014)**	**Gentile et al. (2011)**	**Ko et al. (2014)**	**Ko et al. (2014)**	**(Lau et al., 2017)**	**Scharkow et al. (2014)**	**Strittmatter et al. (2016)**	**Thege et al. (2015)**	**Van Rooij et al. (2011)**
Year of publication	2014	2011	2014	2007	2017	2014	2016	2015	2011
Journal	Addictive behaviors	Pediatrics	Comprehensive psychiatry	Cyberpsychology and behavior	Addictive behaviors	Addiction	Eur. Child and adolescent psychiatry	BMC psychiatry	Addiction
Focus	General internet use	Gaming	General internet use	General internet use	general internet use	Gaming	General internet use	Gaming, online chat	Gaming
Scale employed	CIAS	Gentile	CIAS	CIAS	CIAS	GAS	YDQ	GAPGM	CIUS
Yrs. data collection	2010–2011	2007–2009	2005–2006	2003–2004	2012–2014 (1 year)	2011–2013	2010–2012	2006–2011	2008–2009
Country	Taiwan	Singapore	Taiwan	Taiwan	China	Germany	Germany	Canada	Netherlands
Sample: Regional/National	Regional	National	Regional	Regional	Regional	National	Regional	Regional	National
Setting	Classroom	Classroom	Classroom	Classroom	Classroom	telephone	Classroom + Computer	telephone	Classroom
Age of respondents	15–17	9–14	12–13	12–16	~12–16	14–40+	13–17	Mean age: 46 +/– 14	13–16
Gender (% male at baseline)	48%	73%	50%	52%	54%	Female: 41–44%	48.00%	45% male	49% male
T1 sample size	2,992	2,998	2,293	517	9,666	4,500	1,444	4,121	1,572
Follow-up rate (final data coll.)	77.00%	84%	66%	91%	86%	20%	36%	93%	94%
Prevalence T1	26%	9.9%	9%	18%	16%	Problematic: 3.7% addiction: 0.3%	4.3 %	Online chat: 1.5% video gaming: 1.7%	Of entire population of 5+-16 year olds in Netherlands: 1.6% gamers only: ~3%
Prevalence T1 + 1 year	27%	8.8%	12%	15%	18%	Problematic: 3.2% addiction: 0.3%	27%	Online chat: 1,1% video gaming: 1,4%	1.50%
Persistence T1 + 1 year	62%	84% (2-year)	49%	51%	54%	35%	32% (2-year)	24%	50%

#### Characteristics of the Samples

All of the included studies used samples from Asian or Western populations. Three studies come from Taiwan (Ko et al., 2007, 2014; Chang et al., 2014), one from Hong Kong, China (Lau et al., 2017), and one draws its sample from Singapore (Gentile et al., 2011). Germany has two studies (Scharkow et al., 2014; Strittmatter et al., 2016). One study is from the Netherlands (Van Rooij et al., 2011). One study used a Canadian sample (Thege et al., 2015). The sample strategies differed slightly, where regional samples were most common. Only three studies used national samples (Gentile et al., 2011; Van Rooij et al., 2011; Scharkow et al., 2014). The settings were either classroom or telephone surveys (see Table 1). Table 1 shows that prevalence estimates vary between 1 and 26%, with a non-weighted average of 10%. Prevalence for all studies are included in Table 1.

#### Narrative Review

Six different scales were used in the nine studies. The main contents of the scales are shown in Table 2. The diagnostic criteria largely overlap, using criteria similar to those of the ICD-11 (World Health Organization, 2018), and DSM-5 (American Psychiatric Association, 2013) for gaming disorder, and DSM-IV (Frances, 1994) for pathological gambling. All scales include the following three criteria: Trouble limiting use, Withdrawal, External Problems/Conflict.

**Table 2:** Contents of scales.

**Symptom**	**Scale**					
	**CIAS**	**CIUS**	**GAS**	**Gentile**	**YDQ**	**PAPG/BAM**
Preoccupation/Salience		x	x	X	x	X
Tolerance	x		x	X	x	X
Trouble limiting use	x	x	x	X	x	X
Withdrawal	x	x	x	X	x	X
External problems/conflict	x	x	x	X	x	X
Mood modification/Coping		x	x	X	x	
Failed time management	x		x		x	X
Lying about use					x	

Although similar in their content, the scales vary in their cut-offs: In the most used, CIAS (Chen Internet Addiction Scale, 63–64 out of 104 points are required to be considered as addicted. In the Gentile scale, 5/10 suffices, and in the YDQ (Young Diagnostic Questionnaire) it is 5/8. Van Rooij et al. (2011) employed the CIUS (Compulsive Internet Use Scale) in combination with the amount of time spent per week, using latent class analysis where the cut-off is unclear. However, the mean score in CIUS for the addicted class was 2.9/5. Thege et al. (2015) used a combination of a qualifying question about whether over-involvement in the activity has caused significant problems in the past year, along with PAPG/BAM (Problem and Pathological Gambling Measure/Behavioral Addiction Measure), a measure used to establish the severity of the problem. GAS (Game Addiction Scale), used in Scharkow et al. (2014), required 7/7 criteria for addiction but only 4/7 for problematic use, thus having fewer respondents fulfilling requirements for addiction.

When Chang et al. (2014) compared dropouts from the study to the follow-up group, internet addiction, alcohol drinking and smoking was more common among the dropouts. Lau et al. (2017), found several significant differences: lower school form, lower cues to action, and self-efficacy for reducing internet use, higher probability of depression and loneliness, and having a father with a college education or higher. Strittmatter et al. (2016) found non-respondents at follow-up to more often be male, older and not living with biological parents or relatives, spending more time using their computers for non-academic tasks such as gaming, along with conduct problems, lower pro-social behavior, and showed fewer emotional symptoms. On average, their parents also had less control over their children's activities in their spare time. Thege et al. (2015) found that those missing at least one data collection also reported higher levels of excessive video gaming.

Gentile et al. (2011) did not find differences between dropouts and completers in terms of pathological gaming symptoms. Similarly, Scharkow et al. (2014) found non-specified differences in demographics among the drop-outs, but none in gaming frequency nor problematic game use. Van Rooij et al. (2011) attributed dropouts mostly to whole school classes leaving the study.

Table 1 presents the reported ages in the included studies. All studies but one (Thege et al., 2015) used samples with adolescents, Gentile et al. (2011) also included children, and Scharkow et al. (2014) also included adults. Thus, most ages are covered in this review. Scharkow et al. (2014) found a higher prevalence of problematic gaming among adolescents than among adults. The studies which include adults (Scharkow et al., 2014; Thege et al., 2015) are among the studies with the lowest prevalence.

Most studies had balanced proportions between genders in their samples (50 ± 5% males), except for one (Gentile et al., 2011), where the sample was dominated by boys (73%). Three studies (Ko et al., 2007, 2014; Chang et al., 2014) found boys to be more likely to have an internet addiction, both persistent, and transitory. Gentile et al. (2011) found boys to play more games on average, as well as endorse more symptoms and fulfill pathological gaming criteria at each time. No difference between genders in persistence was presented. Lau et al. (2017) found a mild in tendency for boys be in remission of internet addiction at T2. Strittmatter et al. (2016) did not find any gender differences, but suspected non-response bias from problematic boys. Their sample contained a majority of females. Thege et al. (2015) found problematic video gaming to be more common among the male part of the sample in three of the five waves, although differences in the structure of persistence between genders were not specified.

Chang et al. (2014) found that students who had internet addiction at T1, and an increase in depression and alcohol use, were more likely to persist. Gentile et al. (2011) found that children who persisted as pathological gamers had higher levels of anxiety, social phobia, and depression at T3 as compared to those in remission, as well as lower empathy scores, higher impulsivity, violent game exposure, physically aggressive behavior score, and relationally aggressive behavior score. Ko et al. (2014) presented results showing that scores for hostility, depression, and social phobia decreased more in the group of adolescents who were in remission from internet addiction at T2, than for those with persistent internet addiction. Lau et al. (2017) identified that remission was correlated with lowered scores on various psychometric scales such as loneliness, depression, and social anxiety, and improved scores on positive factors such as family support, self-esteem and positive affect. The results were the opposite for those who reported symptoms for internet addiction at both T1 and T2.

Three studies controlled for some socioeconomic variables. Chang et al. (2014) found that internet addiction at any time of data collection, as well as persistent internet addiction, was a little more common among respondents where fathers', as well as mothers' highest education was high school or lower, as compared to parents with higher education. Similarly, in households with poverty as well, internet addiction was more common as well as more persistent. Lau et al. (2017) found no socio-demographic factors to predict remission from internet addiction. Strittmatter et al. (2016) did not find parental employment, nor the child living with its biological parent to matter for pathologic internet use.

### Meta-analysis

#### Overall Persistence

**Figure 2:**
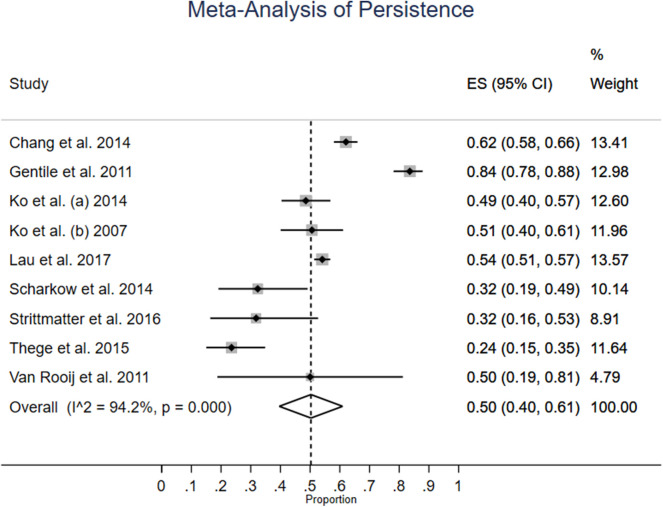
Meta-analysis of persistence.

Figure 2 shows the meta-analysis of persistence rates in all nine studies. The weighted average persistence is 50% (CI: 40–61%). The non-weighted average was 55%. Statistical heterogeneity between studies is very high, at 94.2%, and two studies have confidence intervals which do not overlap with the confidence interval of the weighted estimate. The difference between largest and smallest reported persistence is large: Gentile et al. (2011) reported 84%, while Thege et al. (2015) only had 24%.

### Subgroup Analysis

#### Origin

**Figure 3:**
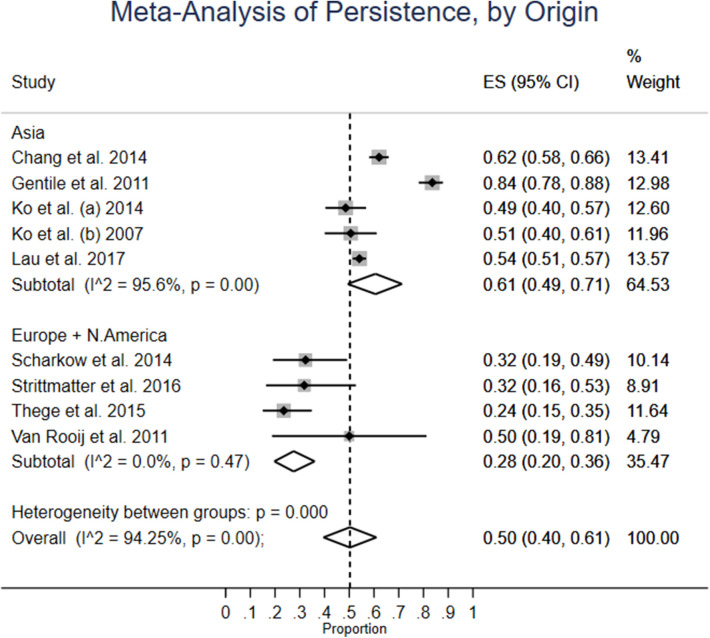
Meta-analysis of persistence, by origin.

Figure 3 organizes the studies' persistence averages in two categories. In the Asian group, the average is 61% (CI: 49–71%), with high heterogeneity: 95.6%. Heterogeneity between groups is significant. The Europe + North America had a weighted average of 28% persistence (CI: 20–36%) and no significant heterogeneity. However, the lower statistical heterogeneity is shadowed a bit when one considers that confidence intervals are large.

#### Scale

**Figure 4:**
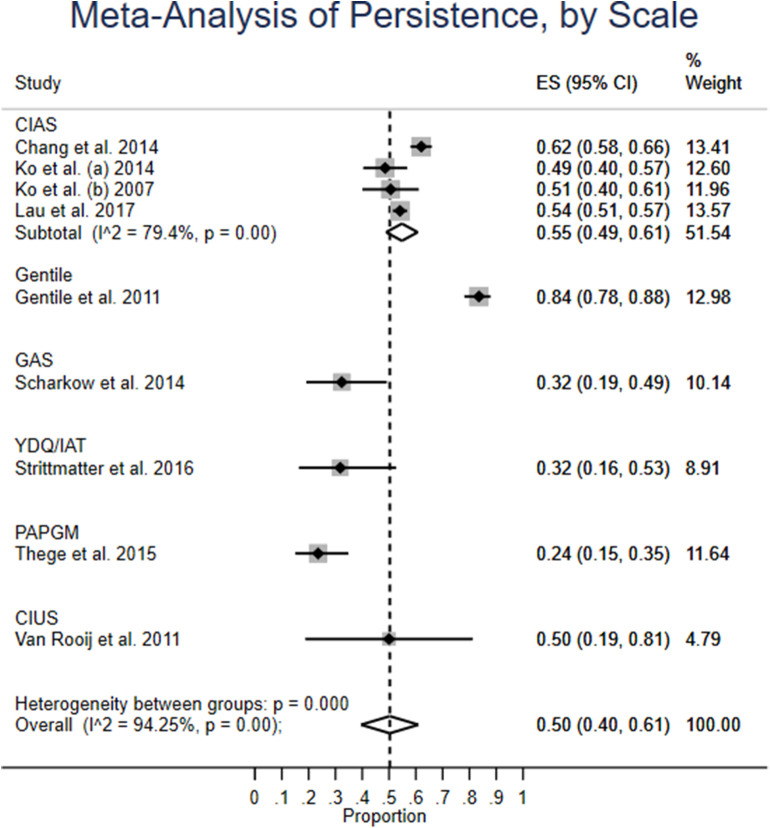
Meta-analysis of persistence, by scale.

The four studies using CIAS have weighted average of 55% (CI: 49–61%) persistence. Heterogeneity is high, but as compared to the other models so far, lower, at 79%. The overall heterogeneity between groups is significant (Figure 4).

#### Focus

**Figure 5:**
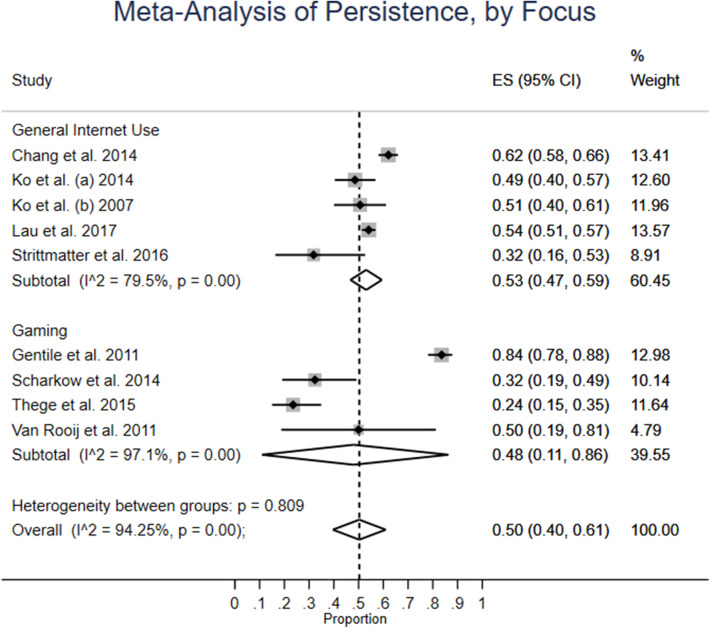
Meta-analysis of persistence, by focus.

In Figure 5, it is shown that four studies study gaming only, while five focus on general internet use. For the gaming-focused group of studies, persistence has a weighted average of 48%, and the confidence interval covers almost the whole possible spectrum: 11–86%. For the general internet use group, the average weighted persistence is close, but much more concentrated: 53% (CI: 47–59%), although there is lower inter-study heterogeneity: 79.5% than for gaming-only (97.1%). There is no statistically significant heterogeneity between the two groups, meaning that there is no difference in persistence.

#### Correlation Between Prevalence and Persistence

There seems to be a positive relationship between prevalence and persistence. As estimated by Pearson's r, the correlation is 0.46, with a *p* < 0.21. However, Gentile et al. (2011) present an unusual combination of a fairly low prevalence estimate and a high persistence estimate. If that study is removed, the correlation dramatically rises to 0.8 and the *p* < 0.02.

## Discussion

The research objective for this systematic review and meta-analysis was to explore whether there is evidence for the view of problematic internet use as a persistent condition. Nine peer-reviewed studies were included, all published after 2007. One-year persistence varied between 24 and 84%, with a weighted average of 50% (non-weighted average: 55%). Persistence of problematic internet use was tested using random-effects inverse variance meta-analysis. According to Mihara and Higuchi (2017), the natural course of internet gaming disorder was hard to define, a statement remaining true. However, this systematic review intended to dig deeper into the particularities of these ambiguities, by performing a meta-analysis, complemented by an investigation into whether potential patterns in results could be found when to the studies' scale used, where data was collected or if they focused on gaming or a broader construct like internet addiction. Below follows a discussion of the findings.

Prevalence levels varied between 1 and 26%. Subgroup analysis was performed to investigate the heterogeneity between studies' persistence. Nine studies included in the review used samples from Asia, Europe, or North America. The average level for persistence was lower for the Western countries: 28% as compared to Asia: 61%. This complicates the findings in Mihara and Higuchi's (2017) review, where accounting for prevalence, they did not find regional differences. This may be accredited to the small sample of studies included in the meta-analysis, leaving it sensitive to single studies' findings. However, the wide range of prevalence, as well as the dominance of European studies in Mihara and Higuchi's (2017) review makes for uncertainty in conclusions about general tendencies among the findings of included studies.

Five scales were used in the nine studies, and were largely similar in the diagnostic criteria employed. Three criteria were part of all scales: trouble limiting use, withdrawal, external problems/conflict. There was some variation in the cut-offs used, and studies measuring the highest prevalence rate had generous cut-offs. The study with the most generous cut-off (Gentile et al., 2011) also had the highest persistence, but a prevalence level close to the mean. Precise comparisons between the different scales' cut-offs was limited due to the varying ways of applying them. The logic, however, is clear: When diagnostic scales' contents are comparable, generous cut-offs will lead to catching more individuals, resulting in larger internal heterogeneity: individuals with the same diagnosed problem can have fewer traits in common. In longitudinal assessments, this also means that one can stop having one trait, while fulfilling another and thus keep the same score on the scale.

There is a positive relationship between prevalence and persistence estimates. It makes logical sense, since persistence is a proportion of prevalence, and it would be likely that the same aspects of study design that affect the prevalence estimate will also affect persistence. This finding could be useful for hypothesizing in future studies: high prevalence estimates seem to produce high persistence estimates. The amount of people forming the basis for analysis is not large: In total, 2,438 individuals form the “prevalence” group, and 1,385 people make up the “persistence” group. In one study by Van Rooij et al. (2011), the addicted class of participants was six in the first wave in and three in the final wave. Similarly, all European and North American samples had persistence groups of <20 people. Thus, one should be careful in using quantitative generalizations. If anything, the results of this review confirm that very little is known about persistence in problematic internet use.

Four studies (Ko et al., 2007, 2014; Chang et al., 2014; Lau et al., 2017) had the same origin, Asia, and overlapped in the other dimensions in which subgroups were tested. These studies also reported high levels of persistence, estimated around 55% (± 6%). Furthermore, all these studies employed regional samples in a school setting. Their similarities in several dimensions make it hard to assess which factor that may be most dominant for the outcome. However, studies using samples in Europe and North America showed wider variation in the different subgroup categories, than did the Asian studies. Across the board, they presented lower prevalence as well as persistence, except for one (Van Rooij et al., 2011), where the persistence rate was on par with the Asian studies. The tendency thus seems to be a higher level of problematic internet use in the Asian samples, most prominently Taiwan, than in the European and North American samples, regarding both prevalence and persistence. The mechanism behind this is beyond the limits of this study, but it is possible that different cultures, norms, and lifestyle could be important in further deepening the knowledge about problematic internet use.

Results in the studies (Gentile et al., 2011, Ko et al., 2014, Chang et al., 2014; Lau et al., 2017) controlling for psychosocial measures generally confirmed the significance of personal or psychiatric problems. Van Rooij and Kardefelt-Winther's (2017) worries about surveys systematically missing out on the target population were furthered by the included studies' internal attrition analyses.

Findings support the assumption of problematic internet use as being more prominent among males than females, although not as solely a male phenomenon: some studies reported mild or non-existent differences between genders. Socioeconomic variables are largely neglected. It is remarkable that a factor so well-established as important for health and economic outcomes is largely neglected in this field of research.

### Conclusions

Results of this systematic review and meta-analysis have shown that it is difficult to draw any firm conclusions about the persistence of problematic internet use. Some of these heterogeneity sources are due to active choices made by the authors of this review. More restrictive criteria in e.g., reporting, scale used, or populations, would perhaps have increased comparability, but at the expense of limiting the sources of evidence.

The first part of the review consisted of a literature search. Only nine studies of sufficient quality were found, and only two more studies provided sufficient information on persistence to even be considered for quality assessment. This finding itself indicates that persistence in problematic internet use is an under-researched topic. There are many possible explanations for this. A pragmatic reason could be that panel surveys are costly to perform, and it may be difficult to get funding for a problem that up until recently lacked formal recognition.

All studies found some individuals that showed persistent symptoms 1 year after the first assessment. This group should be a focus for future research, and more knowledge is needed about how they can be distinguished from individuals showing transitory problems. By identifying key traits, persistent problems may be better prevented, or treated. It could also increase the precision of diagnostic tools so that well-functioning heavy gamers do not get falsely identified as problematic.

Future research should aim to understand not only personal motivations and psychosocial correlates behind problematic internet use, but also cultural differences that may explain the different outcomes for Western countries and Asia, and factors in information about class, gender, and age. To increase the precision of diagnosis, future studies should keep investigating heterogeneity as well as comorbidities relating to persistent problems to better tailor prevention, identification, and treatment.

Future policies and public action to limit the harm of problematic internet use should be humble toward the unstable empirical ground. This does not mean that nothing should be done, nor that the research that has already been produced is without merit. Rather, it means that findings that challenge popular descriptions should be acknowledged, and scales, and interpretation should continue to develop to best fit these findings.

## Limitations

Language bias may have shadowed results from non-English speaking countries, although many studies of non-English speaking (Asian) samples were included in the review.

Since this review has had a primary focus on persistence, only studies with a longitudinal study design have been included. Thus, there are longitudinal studies studying psychosocial, gender, socioeconomic, or other relevant contributors to problematic internet use that have been exluded from the review because they did not present sufficient reporting on perstistence. Therefore, this review is not to be seen as a complete recapitulation of these.

## Data Availability Statement

The dataset generated for this study can be obtained upon request to daniel.dahl@sociology.su.se.

## Author Contributions

KB and DD contributed conception and design of the study. DD performed database searches and statistical analysis in agreement with KB. DD wrote first and second draft of the manuscript. Both authors contributed to manuscript revision, read and approved the submitted version.

## Conflict of Interest

The authors declare that the research was conducted in the absence of any commercial or financial relationships that could be construed as a potential conflict of interest.

## Footnotes

^1^In this article, persistence is used to describe a dichotomous state, where individuals fulfill a sufficient amount of criteria to be defined as having or not having a problem, or which in a clinical setting would define if one is qualified to receive care. Thus, this categorical persistence does not account for persistence of specific symptoms or dimensions of assessment instruments.

^2^TS=(“internet gaming disorder” OR “internet dependency” OR “internet overuse” OR cyberaddict^*^ OR “compulsive computer use”) OR TS=((Internet OR “internet gaming” OR “internet game” OR cyberspace OR online OR net OR “cyber game” OR technology OR “video game” OR virtual OR “social media” OR sns OR “online network” OR “online networking” OR computer OR smartphone OR cyber) NEAR/1 addict^*^) OR TS=((problem^*^ OR pathologic^*^ OR compuls^*^ OR excessive OR disorder^*^ OR unregulat^*^) NEAR/1 “internet use”) AND TS= (longitud^*^ OR persist^*^ OR “over time” OR endur^*^ OR “follow-up” OR panel OR “time series”).
